# HDL cholesterol is associated with PBMC expression of genes involved in HDL metabolism and atherogenesis

**DOI:** 10.2478/jomb-2019-0052

**Published:** 2020-09-02

**Authors:** Liudmila V. Dergunova, Elena V. Nosova, Veronika G. Dmitrieva, Alexandra V. Rozhkova, Ekaterina V. Bazaeva, Svetlana A. Limborska, Alexander D. Dergunov

**Affiliations:** 1 Institute of Molecular Genetics of the Russian Academy of Sciences, Laboratory of Functional Genomics, Moscow, Russia; 2 National Research Centre for Preventive Medicine, Laboratory of Structural Fundamentals of Lipoprotein Metabolism, Moscow, Russia

**Keywords:** atherogenesis, gene expression, HDL functionality, HDL and atherogenesis-prone genes, human PBMC, aterogeneza, ekspresija gena, HDL funkcionalnost, HDL i geni skloni aterogenezi, humani PBMC

## Abstract

**Background:**

To reveal the association of plasma level of high density lipoprotein cholesterol (HDL-C) level with the transcript level of annotated genes in peripheral blood mononuclear cells (PBMC) and involved in HDL metabolism and atherogenesis at the absence of morphologically evident coronary stenosis.

**Methods:**

Transcript levels of 63 genes in PBMC from 38 male patients 40-60 years without coronary atherosclerosis with widely varied HDL-C level were measured. The protein interactions were analyzed with STRING database.

**Results:**

Among 22 HDL-related genes, the transcript levels for 10 genes (*ABCA1, BMP1, CUBN, HDLBP, LCAT, LDLR, PRKACB, PRKACG, SCARB1* and *ZDHHC8*) negatively correlated with HDL-C, while positively for APOA1 gene. Among 41 atherosclerosis-prone genes, the transcript levels for 11 genes (*CSF1R, CSF2RB, IL18R1, ITGAM, ITGB3, PRKCQ, SREBF1, TLR5, TLR8, TNFRSF1A* and *TNFRSF1B*) negatively correlated with HDL-C only, not with LDL-C and plasma TG. The protein products efficiently interacted within each cluster while only two intersection nodes existed between clusters.

**Conclusions:**

Coordinate regulation of cholesterol influx and efflux in PBMC in atherosclerosis-free subjects with widely varied HDL-C level is suggested. The decreased synthesis and transport of cholesteryl ester to the liver may contribute to hyperalphalipoproteinemia. HDL-C increase is associated with the decrease of expression of innate immunity and inflammation genes. Visualization of 22 responder genes is suggested to be useful in the validation of HDL functionality and atherogenesis even at the absence of morphologically evident coronary stenosis.

## Abstract (sr)

**Uvod:**

Cilj rada je otkrivanje povezanosti nivoa lipoproteinskog holesterola visoke gustine u plazmi (HDL-C) sa nivoom transkripta anotiranih gena u mononuklearnim ćelijama periferne krvi (PBMC), a koji su uključeni u HDL metabolizam i aterogenezu u odsustvu morfološki evidentne koronarne stenoze.

**Metode:**

Izmereni su nivoi transkripta 63 gena u PBMC kod 38 pacijenata muškog pola starosti između 40 i 60 godina bez koronarne ateroskleroze sa značajno raznovrsnim nivoom HDL-C. Interakcije proteina su analizirane pomoću STRING baze podataka.

**Rezultati:**

Među 22 gena povezana sa HDL, nivoi transkripta za 10 gena (ABCA1, BMP1, CUBN, HDLBP, LCAT, LDLR, PRKACB, PRKACG, SCARB1 i ZDHHC8) su bili u negativnoj korelaciji sa HDL-C, dok je korelacija pozitivna za GEN APOA1. Od 41 gena koji su skloni aterosklerozi, nivoi transkripta za 11 gena (*CSF1R, CSF2RB, IL18R1, ITGAM, ITGB3, PRKCK, SREBF1, TLR5, TLR8, TNFRSF1A* i *TNFRSF1B*) su u negativnoj korelaciji samo sa HDL-C, ali ne sa LL-C-C, i TG u plazmi. Proteinski proizvodi efikasno su delovali u svakom klasteru, dok su između klastera postojala samo dva čvora preseka.

**Zaključak:**

Predlaže se koordinisana regulacija priliva i odliva holesterola u PBMC kod subjekata bez ateroskleroze sa širokim opsegom vrednosti nivoa HDL-C. Smanjena sinteza i transport holesterol estra do jetre može doprineti hiperalfalipoproteinemiji. Povećanje HDL-C povezano je sa smanjenjem ekspresije gena u vezi sa urođenim imunitetom i upalnim procesima. Vizualizacija ovih 22 gena može biti korisna u validaciji HDL funkcionalnosti i aterogeneze, čak i u odsustvu morfološki vidljive koronarne stenoze.

## Introduction

Coronary heart disease is one of the major mortality causes around the globe [1]. The major underlying effects are the disturbances of lipid metabolism and atherogenesis with the contribution of immunological and metabolic processes [2]. For a long time, the primary goal in the treatment of atherogenic dyslipidemia was to decrease the low-density lipoprotein cholesterol (LDL-C) and to increase high-density lipoprotein cholesterol (HDL-C). The proatherogenic role of LDL was opposed to the atheroprotective role of HDL as cholesterol acceptors [3], thus forming the reverse cholesterol transport (RCT) from lipid-loaded macrophages to the liver [4]. Also, HDL possesses many pleiotropic atheroprotective properties such as endothelium protection, antioxidant, anti-inflammatory, anti-apoptotic and anti-thrombotic effects [5] [6]. However, some genetic and clinical data challenged the atheroprotective significance of circulating HDL. The functional mutations of *ABCA1* gene with low HDL-C levels did not increase the risk of coronary heart disease [7], while the increase of HDL-C level did not decrease the risk [8] and even increased it in some circumstances. GWAS studies revealed many single nucleotide polymorphisms associated with HDL content without any definitive conclusions on the association of HDL-C with coronary heart disease [9]. The loss of the atheroprotective properties of HDL may be related to their functional properties, not with their concentration [10]. The appearance of dysfunctional HDL may be caused by the loss of anti-inflammatory and antioxidant proteins with the concomitant appearance of pro-inflammatory proteins in HDL particles [11] [12]. Thus, the epidemiology, genetics, clinical and experimental data accumulated up to date do not unequivocally relate HDL-C as an anti-atherogenic factor.

To reveal the HDL role on the transcriptome level, we selected some genes with the expression in peripheral blood mononuclear cells (PBMC) [13]. Among them, 22 genes involved in HDL metabolism (HDL cluster) were chosen. In addition, 41 genes involved in inflammation and other atherogenesisprone processes (atherogene cluster) were included based on the GWAS data on differentially expressed genes in PBMC in atherosclerosis versus control and RT-PCR data [13]. In a pilot study with a small group of patients without coronary atherosclerosis, we com-pared the expression of HDL cluster genes in PBMC, HDL charge heterogeneity and cholesterol acceptor capacity of patient HDL in cholesterol efflux assay [14]. The expression of several HDL-related genes, cholesterol efflux efficiency and concentration of preb-HDL significantly differed between hypo-and hyperalphalipoproteinemics [14]. However, the joint consideration of the expression of atheroprotective and pro-atherogenic genes in relation to HDL-C level in the same group of patients is still lacking.

The aim of the present study is to reveal the association of plasma level of high-density lipoprotein cholesterol level with the transcript level of selected genes in peripheral blood mononuclear cells (PBMC) and involved in HDL metabolism and atherogenesis at the absence of morphologically evident coronary stenosis.

## Materials and Methods

### Patients and laboratory tests

Thirty-eight white Caucasian male patients at the age between 40 and 60 from the Moscow region were enrolled in the study (Table 1). The inclusion criteria were the absence of coronary deficiency and stenosis of coronary/carotid arteries verified by coronary angiography and ultrasonography, respectively, the absence of hypertension and diabetes, non-alcoholics, the absence of therapy by corticosteroids and hypolipidemic agents for at least 3 months. The informed consent was obtained from each patient, and the local ethical committee approved protocol corresponding to the Helsinki declaration from 1975. Plasma lipid levels (Table 1) were measured by enzyme methods with Architect c8000 (Abbott, USA).

**Table 1:** Characteristics of the study group. White Caucasian male patients from the Moscow region were enrolled in the study. Parameter variations between minimal and maximal values are given also.

	Mean ± SD (n = 38)	Variation
Age, years	49.1 ± 5.7	40.0 – 60.0
Body mass index, kg/m^2^	28.5 ± 3.1	21.0 – 31.0
Chol, mmol/L	5.19 ± 1.27	2.40 – 7.80
HDL-C, mmol/L	1.19 ± 0.35	0.59 – 2.24
LDL-C, mmol/L	3.31 ± 1.05	1.14 – 5.98
TG, mmol/L	1.52 ± 0.71	0.59 – 3.42

### Gene expression analysis by quantitative real-time PCR

Ficoll-Hypaque (1.077 g/mL) density gradient centrifugation (Sigma, USA) was performed to isolate peripheral blood mononuclear cells. Total RNA was isolated with TRI Reagent (Molecular Research Center, USA) and traces of DNA were removed by DNase I in the presence of RNase inhibitor according to the manufacturer's protocol (ThermoFisher Scientific, USA). RNA samples were kept at -70 °C. RNA concentration and quality (RQI > 9) were measured with the Experion electrophoresis system (Bio-Rad, USA). cDNA was synthesized with RevertAid First Strand cDNA Synthesis Kit (ThermoFisher Scientific, USA) and cDNA samples were kept at -20 °C.

HDL cluster included the following genes: Albumin (*ALB*); Alpha-2-macroglobulin (*A2M*); Amnion associated transmembrane protein (*AMN*); Apolipoprotein A1 (*APOA1*); Apolipoprotein C2 (*APOC2*); Apolipoprotein E (*APOE*); ATP binding cassette subfamily A member 1 (*ABCA1*); ATP binding cassette subfamily G member 1 (*ABCG1*); Bone morphogenetic protein 1 (*BMP1*); Cholesteryl ester transfer protein (*CETP*); Cubilin (*CUBN*); High density lipoprotein binding protein (*HDLBP*); Lecithin-cholesterol acyltransferase (*LCAT*); Lipase C, hepatic type (*LIPC*); Lipoprotein lipase (*LPL*); Low density lipoprotein receptor (*LDLR*); Phospholipid transfer protein (*PLTP*); Protein kinase cAMP-activated catalytic subunit alpha (*PRKACA*); Protein kinase cAMP-activated catalytic subunit beta (*PRKACB*); Protein kinase cAMP-activated catalytic subunit gamma (*PRKACG*); Scavenger receptor class B member 1 (*SCARB1*); Zinc finger DHHC-type containing 8 (*ZDHHC8*).

Atherogene cluster included the following genes: Asialoglycoprotein receptor 2 (*ASGR2*); CD14 molecule (*CD14*); CD36 molecule (*CD36*); Coagulation factor V (*F5*); Colony stimulating factor 1 receptor (*CSF1R*); Colony stimulating factor 2 receptor beta common subunit (*CSF2RB*); C-X-C motif chemokine ligand 5 (*CXCL5*); Cytochrome b-245 alpha chain (*CYBA*); Integrin subunit alpha 2b (*ITGA2B*); Integrin subunit alpha M (*ITGAM*); Integrin subunit beta 3 (*ITGB3*); Intercellular adhesion molecule 1 (*ICAM1*); Interleukin 1 beta (*IL1B*); Interleukin 1 receptor type 1 (*IL1R1*); Interleukin 18 (*IL18*); Interleukin 18 receptor 1 (*IL18R1*); Interleukin 18 receptor accessory protein (*IL18RAP*); Junctional adhesion molecule 3 (JAM3); Lymphotoxin alpha (*LTA*); Matrix metallopeptidase 9 (*MMP9*); Microsomal glutathione S-transferase 1 (*MGST1*); NPC intracellular cholesterol transporter 1 (*NPC1*); NPC intracellular cholesterol transporter 2 (*NPC2*); Nuclear receptor subfamily 1 group H member 2 (*NR1H2*); Nuclear receptor subfamily 1 group H member 3 (*NR1H3*); Oxidized low density lipoprotein receptor 1 (*OLR1*); Phosphatidylcholine transfer protein (*PCTP*); Phospholipase A2 group VII (*PLA2G7*); Protein kinase C theta (*PRKCQ*); S100 calcium binding protein A12 (*S100A12*); S100 calcium binding protein A8 (*S100A8*); S100 calcium binding protein A9 (*S100A9*); Secretory leukocyte peptidase inhibitor (*SLPI*); Solute carrier family 7 member 11 (*SLC7A11*); Sterol regulatory element binding transcription factor 1 (*SREBF1*); Superoxide dismutase 2 (*SOD2*); TNF receptor superfamily member 1A (*TNFRSF1A*); TNF receptor superfamily member 1B (*TNFRSF1B*); Toll like receptor 5 (*TLR5*); Toll like receptor 8 (*TLR8*); Vascular endothelial growth factor A (*VEGFA*).

Housekeeping genes included Glyceraldehyde-3-phosphate dehydrogenase (*GAPDH*); Lactate dehydrogenase A (*LDHA*) and Ribosomal protein L3 (*RPL3*) genes.

Gene-specific primers were designed using OLIGO Primer Analysis Software 6.31 (Molecular Biology Insights, USA). Primer structures and coordinates according to mRNA for forty-one genes involved in atherogenesis and for two HDL-related LDLR and LIPC genes are given in Table 2. We described earlier the primers for three housekeeping GAPDH, RPL3 and LDHA genes and for twenty HDLrelated genes ABCA1, ABCG1, SCARB1, CETP, PRKACA, PRKACB, PRKACG, LCAT, HDLBP, ZDHHC8, A2M, AMN, ALB, CUBN, BMP1, LPL, PLTP, APOA1, APOE and APOC2 (14). Analysis of primer specificity and protocol of real-time quantitative polymerase chain reaction (qPCR) were performed as previously described [14].

**Table 2:** The primers used for a quantitative real-time polymerase chain reaction *) the data for other twenty HDL-related and three housekeeping genes were published [14]

Primer	GeneBank	5’- 3’ nucleotide sequence	location	efficiency
Genes involved in atherogenesis				
* ASGR2 (F) *	NM_001181.4	GGAGAAACAGCAGCAGGACC	663–682	1.65
* ASGR2 (R) *		GAGTGAGAGAACCAGTAGCAGC	827–848	
* CD14 (F) *	NM_000591.3	CAAGTGTGAAGCCTGGAAGC	277–296	1.80
* CD14 (R) *		ACAAGGTTCTGGCGTGGTC	436–454	
* CD36 (F) *	NM_000072.3	CCTTTGCCTCTCCAGTTGAA	1310–1329	1.99
* CD36 (R) *		GTACACAGGTCTCCCTTCTTTG	1413–1434	
* CSF1R (F) *	NM_005211.3	GGTGGCAGGAAGGTGATGT	836–854	1.71
* CSF1R (R) *		GGTGTTGTTGTGTTGGAGGA	999–1018	
* CSF2RB (F) *	NM_000395.2	ATCCTCCTCTCCAACACCTCC	776–796	1.80
* CSF2RB (R) *		ACCTCCTTCCTCACCTCCCA	1001–1020	
* CXCL5 (F) *	NM_002994.4	GCTGTTGGTGCTGCTGCT	208–225	1.81
* CXCL5 (R) *		CCGTTCTTCAGGGAGGCTAC	389–408	
* CYBA (F) *	NM_000101.3	TTGTGTGCCTGCTGGAGTA	214–232	1.70
* CYBA (R) *		AGTAGGTAGATGCCGCTCG	421–439	
* F5 (F) *	NM_000130.4	CTGGCTGGTGGCTCCTAA	5781–5798	1.57
* F5 (R) *		ATCTTGCTAATCTGGGCTCC	5941–5960	
* JAM3 (F) *	NM_032801.4	TTCCAGAGCCAATCCCAGA	708–726	1.90
* JAM3 (R) *		TCCGCCAATGTTCAGGTC	874–891	
* ICAM1 (F) *	NM_000201.2	GTGACCGTGAATGTGCTCTCC	1727–1747	1.83
* ICAM1 (R) *		GAGGCGTGGCTTGTGTGTT	1895–1913	
* IL1B (F) *	NM_000576.2	TGTCCTGCGTGTTGAAAGATGA	641–662	1.84
* IL1B (R) *		CTGCTTGAGAGGTGCTGATGTA	796–817	
* IL1R1 (F) *	NM_000877.3	TGCTTACTGGAAGTGGAATG	1099–1118	1.76
* IL1R1 (R) *		TGCTGCATCTATACCATGTG	1278–1297	
* IL18 (F) *	NM_001562.3	GACCAAGGAAATCGGCCT	395–412	1.81
* IL18 (R) *		CACAGAGATAGTTACAGCCATACC	503–526	
* IL18RAP (F) *	NM_003853.3	ACAACCCAGTCCGTCCAAC	1524–1542	1.86
* IL18RAP (R) *		ACATCAGGAAATAGGCTCAGG	1790–1810	
* IL18R1 (F) *	NM_003855.3	CGATAAAGAAGAACGCCGAGT	655–675	1.68
* IL18R1 (R) *		GCAGAGCAGTTGAGCCTTACG	842–862	
*ITGAM (F) *	NM_001145808.1	CTCTCTCCCAGGCTCCAGT	1827–1845	1.79
* ITGAM (R) *		CATTCCTTGCCACTTCCCT	1986–2004	
* ITGA2B (F) *	NM_000419.4	AAGATTGTGCTGCTGGACG	2421–2439	1.81
* ITGA2B (R) *		GAAGGTGGATGCTGAGGTGA	2612–2631	
* ITGB3 (F) *	NM_000212.2	AGTAACCTGCGGATTGGCTT	537–556	1.81
* ITGB3 (R) *		CACACTCTGCTTCTTCACTTCC	713–734	
* LTA (F) *	NM_001159740.2	GCTGCTGCTGGTTCTGCT	282–299	1.65
* LTA (R) *		GTTCTGCTTGCTGGGGTCT	420–438	
* MGST1 (F) *	NM_145792.2	CAGGTAATGGATGATGAAGTA	81–101	1.79
* MGST1 (R) *		GCCAAATGCTACACAGTCTTCT	206–227	
* MMP9 (F) *	NM_004994.2	ACCCTTGTGCTCTTCCCTG	98–116	1.70
* MMP9 (R) *		CGACTCTCCACGCATCTCTG	192–211	
* NPC1 (F) *	NM_000271.4	CAGCCACATAACCAGAGCGT	3778–3797	1.78
* NPC1 (R) *		AGCCAACACCACAATCCCT	3898–3916	
*NPC2 (F)*	NM_006432.3	TCCCATTCCTGAGCCTGAT	362–380	1.82
*NPC2 (R)*		GTTGCCACTCCACCACCA	478–495	
*NR1H2 (F)*	NM_007121.5	GCCATCATCTCAGTCCAGG	1104–1122	1.79
*NR1H2 (R)*		ACTCTGTCTCGTGGTTGTAGC	1237–1257	
*NR1H3 (F)*	NM_005693.3	GCCTTGCTCATTGCTATCAG	1319–1338	1.86
*NR1H3 (R)*		GTGGGAACATCAGTCGGTCA	1447–1466	
*OLR1 (F)*	NM_002543.3	TTGCCTGGGATTAGTAGTGACC	249–270	1.77
*OLR1 (R)*		CTTCTTCTGCTTGTTGCCG	376–394	
*PCTP (F)*	NM_021213.3	GGTGAAGCAATACAAGCAGAG	578–598	2.18
*PCTP (R)*		TAATGAGCCAGGACGGAAT	672–690	
*PLA2G7 (F)*	NM_005084.3	GGCATTGACCTGGCATCTC	759–777	1.86
*PLA2G7 (R)*		TGTGTCTCCTCCTCTTGTTTCAG	894–916	
*PRKCQ (F)*	NM_006257.4	ACTGCCACCTTCTTCCCAC	587–605	1.55
*PRKCQ (R)*		CTTGAGTCCTTGCCGTGC	866–883	
*SLC7A11 (F)*	NM_014331.3	GTCCGCAAGCACACTCCT	1358–1375	1.97
*SLC7A11 (R)*		ATGACGAAGCCAATCCCTG	1629–1647	
*SLPI (F)*	NM_003064.3	CCTTCCTGGTGCTGCTTG	44–61	2.28
*SLPI (R)*		GACAACATCTCTTCTTCCCTGG	193–214	
*SOD2 (F)*	NM_000636.3	CACCACAGCAAGCACCAC	309–326	1.80
*SOD2 (R)*		GTTCTCCACCACCGTTAGG	480–498	
*SREBF1 (F)*	NM_001005291.2	CCTCAGATACCACCAGCGTC	1828–1847	1.68
*SREBF1 (R)*		TTGCGATGCCTCCAGAAGT	2026–2044	
*S100A8 (F)*	NM_001319196.1	ATGCCGTCTACAGGGATGA	267–285	1.74
*S100A8 (R)*		ACGCCCATCTTTATCACCAG	407–426	
*S100A9 (F)*	NM_002965.3	GACCATCATCAACACCTTCCAC	82–103	1.68
*S100A9 (R)*		TAGCCTCGCCATCAGCAT	284–301	
*S100A12 (F)*	NM_005621.1	ATTAGGCTGGGAAGATGACAA	55–75	1.90
*S100A12 (R)*		GTGGGTGTGGTAATGGGCA	320–338	
*TLR5 (F)*	NM_003268.5	GACCCTCTGCCCCTAGAATAA	581–601	1.75
*TLR5 (R)*		GCCATCAAAGGAGCAGGAA	707–725	
*TLR8 (F)*	NM_016610.3	GGAACATCAGCAAGACCCATC	115–135	1.80
*TLR8 (R)*		CGCATAACTCACAGGAACCAGA	279–300	
*TNFRSF1A (F)*	NM_001065.3	GCTCCTTCACCGCTTCAGA	563–581	1.74
*TNFRSF1A (R)*		GGTCCCATTGAGGCAGAGG	744–762	
*TNFRSF1B (F)*	NM_001066.2	AACACACGCAGCCAACTCC	763–781	1.77
*TNFRSF1B (R)*		GTCACACCCACAATCAGTCCA	875–895	
*VEGFA (F)*	NM_001025366.2	GAGGGCAGAATCATCACGAA	1136–1155	1.79
*VEGFA (R)*		CATCAGGGGCACACAGGA	1264 – 1281	
HDL-related genes*)				
*LDLR (F)*	NM_000527.4	GAGGTGGCCAGCAATAGAA	1490–1508	2.00
*LDLR (R)*		GATGACGGTGTCATAGGAAGAG	1572–1593	
*LIPC (F)*	NM_000236.2	CTTCAACTCCTCCCTGCCTCT	285–305	1.87
*LIPC (R)*		TGGTGTAGTGGTCGTGGGC	433–451	

### Analysis of interaction between protein products of genes belonging to two clusters

The analysis was performed with STRING database [15] with six kmeans clusters. The p-value for protein-protein interaction (PPI) enrichment is an indicator of interaction efficiency. The number of nodes and the number of edges are network characteristics.

### Statistical analysis

Statistical analysis was performed using STATISTICA 8.0 data analysis software system (StatSoft, Inc. (2008)). We applied the Shapiro-Wilks W test to check the normality and nonnormally distributed data were log-transformed to approach a Gaussian distribution. The data are expressed as mean ± SD. The analysis of primer efficiencies was done with the REST software [16], and the relative expression of all target genes normalized by the geometric mean of three reference genes was used in the correlation analysis. We did linear regression and Pearson’s correlation r tests between biochemical variables with transcript levels to determine the statistical significance and correlation. We used the Benjamini-Hochberg correction for multiple comparisons with the false discovery rate (FDR) value as 0.15 within the recommended range of 0.10 - 0.20 [17]. P-values < 0.05 were considered statistically significant.

## Results

### Expression of genes related to HDL metabolism

PBMC expression level of 22 genes included in HDL cluster was measured by RT-PCR and significant associations of transcripts with plasma lipids are given in Table 3. Significant associations existed between the transcripts of eleven genes and HDL-C. HDL-C level positively correlated with mRNA content of *APOA1* gene, while negatively with transcripts of cholesterol transporters (*ABCA1 *and* SCARB1*), LDL receptor (*LDLR*), lecithin:cholesterol acyltransferase (*LCAT*), zinc finger DHHC-type containing 8 (*ZDHHC8*), bone morphogenetic protein 1 (*BMP1*), high density lipoprotein binding protein (*HDLBP*), cubilin (*CUBN*), protein kinase cAMP-activated catalytic subunit beta and gamma (*PRKACB*, *PRKACG*). Total cholesterol level also negatively correlated with transcript levels of the majority of the above-mentioned genes with the exception of LDLR and PRKACB genes. However, LDL-C level negatively correlated with transcripts of *CUBN* and *HDLBP* genes. Importantly, plasma TG level did not correlate with the transcript levels of HDL cluster genes.

**Table 3:** Bivariate correlations between transcript level of HDL cluster and atherogene cluster genes and lipids. Pearson correlation coefficient and p-values in brackets are included only for significant associations corrected for multiple comparisons by the Benjamini-Hochberg procedure. The functionality of protein products derived from STRING database and literature data is included also.

Gene	HDL-C	Chol	LDL-C	Protein functionality
HDL cluster				
* ABCA1 *	-0.3759 (0.0200)	-0.3906 (0.0150)		cAMP-dependent and sulfonylurea-sensitive anion transporter. Key gatekeeper influencing intracellular cholesterol transport; Belongs to the ABC transporter superfamily. Activated by phosphorylation ATP-bind- ing cassette transporter A1 (ABCA1) interacts with ApoA–I, allowing their lipidation and formation of pre-ß HDL particles that leads to the efflux of free cholesterol and phospholipid from different cell types [18] [19].
* APOA1 *	0.3579 (0.0270)			Participates in the reverse transport of cholesterol from tissues to the liver for excretion by promoting cholesterol efflux from tissues and by acting as a cofactor for the lecithin cholesterol acyltransferase (LCAT). ApoA–I, a mature protein product of *APOA1*, as a part of pre-ß HDL particles binds cellu- lar cholesterol and phospholipids that leads to lipid efflux from the cell [20] [21].
* BMP1 *	-0.4853 (0.0020)	-0.3654 (0.0240)		Cleaves the C-terminal propeptides of procollagen I, II and III. Induces cartilage and bone formation. May participate in dorsoventral patterning during early development by cleaving chordin (CHRD). Responsible for the proteolytic activation of lysyl oxidase LOX. Being the metalloproteinase, BMP-1 leaves human 249-aa proapolipoprotein ApoA–I to generate the mature 243 aa polypeptide and leads to its activation for lipid binding and efflux from the cell as nascent HDL [22]. This conversion is going in the presence of the cell surface lipid transporter ABCA1.
* CUBN *	-0.4282 (0.0070)	-0.4281 (0.0070)	-0.4360 (0.0060)	Cotransporter which plays a role in lipoprotein, vitamin and iron metabolism, by facilitating their uptake. Binds to ALB, MB, Kappa and lambda-light chains, TF, hemoglobin, GC, SCGB1A1, APOA1, high-density lipoprotein, and the GIF-cobalamin complex. The binding of all ligands requires calcium. The multili- gand endocytic receptor Cubilin (*CUBN*), together with its coreceptor, LDL-related protein-2 (LRP2), can bind to apolipoprotein ApoA-I, a major component of HDL, and promote their endocytosis, which leads to their reabsorption into the plasma from glomerular fil- trate, ensuring the maintenance of their plasma levels [23] [24] [25].
*HDLBP*	-0.3540 (0.0320)	-0.4733 (0.0030)	-0.4321 (0.0080)	Vigilin; Appears to play a role in cell sterol meta bo lism. Itmay function to protect cells from over-accumulation ofcholesterol. The actual function of vigilin (*HDLBP*) isunknown. Vigilin induced by cholesterol and sterol hormonesand found human atherosclerotic lesions. The highconstitutive levels of vigilin mRNA in fibroblasts of patientswith familial HDL deficiency may reflect impaired cholesteroltransport in these cells [26] [27].
*LCAT*	-0.3536 (0.0290)	-0.3249 (0.0470)		Synthesized mainly in the liver and secreted into plasmawhere it converts cholesterol and phosphatidylcholines(lecithins) to cholesteryl esters and lysophosphatidylcholineson the surface of high and low-density lipoproteins(HDLs and LDLs). The cholesterol ester is then transportedback to the liver. Lecithin: cholesterolacyltransferase (*LCAT*) promotes the conversion of the discoidal(nascent) HDL to spherical HDL [28] [29]. LCATreacts with the unesterified cholesterol in HDL, transferringthe 2-acyl group of lecithin or phosphatidylethanolamine tothe free hydroxyl residue of cholesterol to generatecholesteryl esters, which are retained in the core of HDL.
*LDLR*	-0.3784 (0.0230)			Binds LDL, the major cholesterol-carrying lipoprotein ofplasma, and transports it into cells by endocytosis. In orderto be internalized, the receptor-ligand complexes must firstcluster into clathrin-coated pits. The low-density lipoproteinreceptor (LDLR) binds cholesterol-rich LDL particlesand promote their clearance from the circulation into thecells where in the lysosomes cholesterol of LDL particlesbecomes available again by hydrolysis of the cholesterylesters [30].
*PRKACB*	-0.3162 (0.0530)	-0.3637 (0.0270)		Mediates cAMP-dependent signalling triggered by receptorbinding to GPCRs. PKA activation regulates diversecellular processes such as cell proliferation, the cell cycle,differentiation and regulation of microtubule dynamics,chromatin condensation and decondensation, nuclearenvelope disassembly and reassembly, as well as regulationof intracellular transport mechanisms and ion flux.Being the catalytic subunit of cAMP-dependent proteinkinase (PKA), the protein product of *PRKACB* is involvedin PKA-mediated ABCA1 phosphorylation, allowing lipidationof ApoA–I which leads to lipid efflux from the cellas nascent HDL [18] [31] [32].
*PRKACG*	-0.3569 (0.0300)	-0.4656 (0.0030)		Phosphorylates a large number of substrates in the cytoplasmand the nucleus; Belongs to the protein kinasesuperfamily. Being the catalytic subunit of cAMP-dependentprotein kinase (PKA), the protein product of*PRKACG* is involved in PKA-mediated ABCA1 phosphorylation,allowing lipidation of ApoA–I which leads to lipidefflux from the cell as nascent HDL [18] [31] [32].
*SCARB1*	-0.4439 (0.0050)			Receptor for different ligands such as phospholipids,cholesterol ester, lipoproteins, phosphatidylserine andapoptotic cells. Receptor for HDL, mediating selectiveuptake of cholesteryl ether and HDL-dependent cholesterolefflux. Also facilitates the flux of free and esterifiedcholesterol between the cell surface and apoB-containinglipoproteins and modified lipoproteins, although lessefficiently than HDL. Scavenger receptor class B type I(*SCARB1*) interacts with spherical and discoidal HDL particlesand remodels the HDL [28].
*ZDHHC8*	-0.4923 (0.0020)			Palmitoyltransferase involved in glutamatergic transmission.Mediates palmitoylation of ABCA1. Palmitoylationof ABCA1 may regulate its function as a transporter andtherefore lipid efflux from the cell [33].
atherogene cluster				
*CSF1R*	-0.3512 (0.0330)			Tyrosine-protein kinase that acts as cell-surface receptor forCSF1 and IL34 and plays an essential role in the regulationof survival, proliferation and differentiation of hematopoieticprecursor cells, especially mononuclear phagocytes, such asmacrophages and monocytes. Promotes the release ofproinflammatory chemokines in response to IL34 andCSF1, and thereby plays an important role in innate immunityand in inflammatory processes.
*IL18R1*	-0.3898 (0.0170)			Within the IL18 receptor complex, responsible for the bindingof the proinflammatory cytokine IL18, but not IL1A norIL1B (Probable). Contributes to IL18-induced cytokine production,either independently of SLC12A3, or as a complexwith SLC12A3.
*ITGB3*	-0.5355 (0.0010)			Integrin alpha-V/beta-3 (ITGAV:ITGB3) is a receptor forcytotactin, fibronectin, laminin, matrix metalloproteinase-2,osteopontin, osteomodulin, prothrombin, thrombospondin,vitronectin and von Willebrand factor. Integrin alpha-IIb/beta-3 (ITGA2B:ITGB3) is a receptor for fibronectin, fibrinogen,plasminogen, prothrombin, thrombospondin andvitronectin. Integrins alpha-IIb/beta-3 and alpha-V/beta-3recognize the sequence R-G-D in a wide array of ligands.Integrin alpha- IIb/beta-3 recognizes the sequence H-H-LG-G-G-A-K-Q-A-G-D-V in fibrinogen gamma chain.
*ITGAM*	-0.4039 (0.0130)			Integrin ITGAM/ITGB2 is implicated in various adhesiveinteractions of monocytes, macrophages and granulocytesas well as in mediating the uptake of complement-coatedparticles. It is identical with CR-3, the receptor for the iC3bfragment of the third complement component. It probablyrecognizes the R-G-D peptide in C3b. IntegrinITGAM/ITGB2 is also a receptor for fibrinogen, factor X andICAM1.
*SREBF1*	-0.3666 (0.0260)			Transcriptional activator required for lipid homeostasis.Regulates transcription of the LDL receptor gene as well asthe fatty acid and to a lesser degree the cholesterol synthesispathway (By similarity). Binds to the sterol regulatory element1 (SRE-1) (5’-ATCACCCCAC-3’).
*TLR5*	-0.3649 (0.0240)			Participates in the innate immune response to microbialagents. Mediates detection of bacterial flagellins. Acts viaMYD88 and TRAF6, leading to NF-kappa-B activation,cytokine secretion and the inflammatory response.
*TLR8*	-0.5129 (0.0010)			Key component of innate and adaptive immunity. TLRs controlhost immune response against pathogens throughrecognition of molecular patterns specific to microorganisms.Acts via MYD88 and TRAF6, leading to NF-kappa-Bactivation, cytokine secretion and the inflammatory.
*TNFRSF1A*	-0.3718 (0.0220)			Receptor for TNFSF2/TNF-alpha and homotrimericTNFSF1/lymphotoxin-alpha. The adapter molecule FADDrecruits caspase-8 to the activated receptor. The resultingdeath-inducing signalling complex (DISC) performs caspase-8 proteolytic activation which initiates the subsequentcascade of caspases (aspartate- specific cysteine proteases)mediating apoptosis.
*TNFRSF1B*	-0.4677 (0.0030)			Receptor with high affinity for TNFSF2/TNF-alpha andapproximately 5-fold lower affinity for homotrimericTNFSF1/lymphotoxin-alpha. The TRAF1/TRAF2 complexrecruits the apoptotic suppressors BIRC2 and BIRC3 toTNFRSF1B/TNFR2. This receptor mediates most of themetabolic effects of TNF-alpha. Isoform 2 blocks TNFalpha-induced apoptosis, which suggests that it regulatesTNF-alpha function by antagonizing its biological activity.
*CSF2RB*	-0.4080 (0.0120)			High affinity receptor for interleukin-3, interleukin-5 andgranulocyte-macrophage colony-stimulating factor.
*PRKCQ*	-0.4077 (0.0140)			Calcium-independent, phospholipid- and diacylglycerol(DAG)-dependent serine/threonine-protein kinase thatmediates non- redundant functions in T-cell receptor (TCR)signalling, including T-cells activation, proliferation, differentiationand survival, by mediating activation of multipletranscription factors such as NF-kappa-B, JUN, NFATC1and NFATC2.

### Expression of atherogenesis-prone genes

HDL-C negatively correlated with the transcript levels of eleven genes among 41 genes included in atherogene cluster (Table 3). These genes code integrin subunit beta 3 (*ITGB3*), Toll-like receptors 5 and 8 (TLR5 and TLR8), TNF receptor superfamily member 1A and 1B (*TNFRSF1A* and *TNFRSF1B*); interleukin 18 receptor 1 (*IL18R1*), integrin subunit alpha M (*ITGAM*); colony-stimulating factor 2 receptor beta common subunit (*CSF2RB*), colony-stimulating factor 1 receptor (*CSF1R*), sterol regulatory element-binding transcription factor 1 (*SREBF1*), and protein kinase C theta (*PRKCQ*).

### Functional interactions between protein products of genes included in two clusters

**Figure 1:**
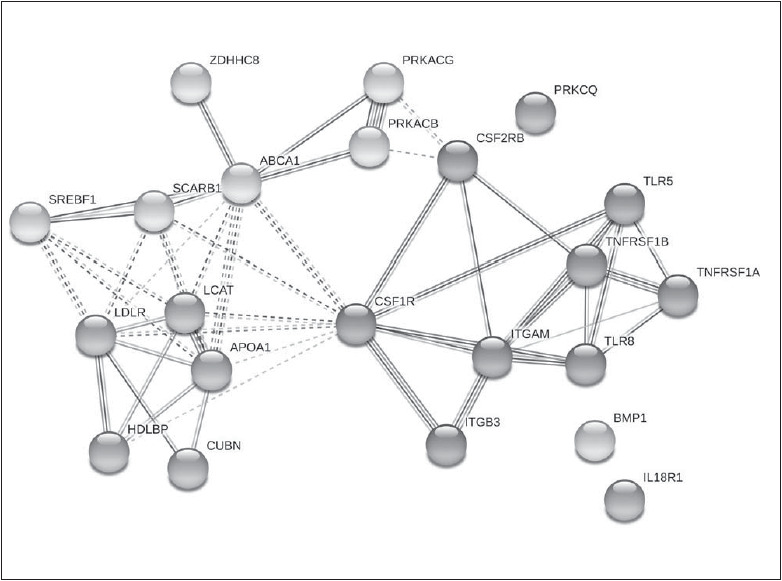
STRING analysis of protein-protein interactions between proteins coded by genes from HDL and atherogene clusters Six kmeans clusters are chosen. Number of nodes: 22; number of edges: 50; average node degree: 4.55; avg. local clustering coefficient: 0.647; expected number of edges: 11; PPI enrichment p-value: < 1.0e-16.

The functional interactions between protein products of 22 genes, with PBMC expression level significantly associated with HDL-C level, were revealed by STRING database [15]. The functional properties of proteins are included in Table 3. Twentytwo nodes are involved in 50 interactions (Figure 1), which significantly exceeds eleven random interactions. Generally, nine from eleven genes from HDL cluster belong to Reactome Plasma lipoprotein assembly, remodelling, and clearance pathway (HSA-174824), based on minimal FDR value mentioned in STRING database. However, nine from eleven genes from the atherogene cluster belong to the Immune System pathway (HSA-168256). Two clusters intersect only at two nodes, namely CSFR1 (six edges) and CSF2RB (two edges) (Figure 1).

## Discussion

The associations between plasma lipids and PBMC expression of 63 genes included in two clusters were studied for a cohort of 38 middle-aged male patients without coronary atherosclerosis with widely varied HDL-C. For the first time, the significant positive correlation between HDL-C and *APOA1* expression and negative correlations of HDL-C and transcripts of ten HDL-related genes and eleven atherogenesis-prone genes even at the absence of morphologically evident coronary stenosis were revealed. The protein products efficiently interacted within both clusters, but only two-node intersections existed between them.

Monocytes are the main source of proinflammatory mediators at atherogenesis [34] that constitute one-fifth of the mononuclear cell fraction. PBMC expression of 41 genes included in the atherogene cluster was measured in the present study for 38 patients with a widely varied HDL-C level. HDL-C level negatively correlated with transcript content for eleven genes. The protein products of the some genes are of primary importance in inflammation and immune response, namely colony-stimulating factor receptors, TNF receptor superfamily members 1A and 1B, TLR5 and TLR8 receptors that induce the proinflammatory cytokine, and IL18 cytokine receptor that participates in innate immunity response and is a critical molecule in atherosclerosis-connected inflammation [35]. Molecular mechanisms of the inhibition of expression by HDL remain unknown. HDL inhibits endothelial and monocyte expression of tissue factor (TF), the major coagulation factor, by inhibiting the activation of p38 MAPK (mitogen-activated protein kinase) and the repression of the PI3K (phosphoinositide 3-kinase) pathway responsible for TF expression [36]. It should be noted that the negative correlation between HDL-C and *ITGB3* and *ITGAM* gene transcripts observed here coincides with the inhibition of integrin and endothelial cell adhesion molecules by HDL [6] [37]. Overall, our data on the negative correlation between HDL-C and pro-inflammatory gene transcripts suggest the decay of atherogenesis-related inflammation with the increase of HDL-C. Besides, PBMC expression of atherogenesis-sensitive genes is suggested to be controlled only by HDL-C level, due to the lack of any association with LDL-C and plasma TG. Both conclusions are the major novelties of our study.

The limitation of our study is the expression profiling of only two gene clusters; the genome-wide transcriptome analysis may reveal some additional genes involved in atherogenesis-related pathways. Also, a detailed study of mRNA and protein expression and enzyme and receptor activities would confirm the metabolic traits suggested to be influenced by the genes in two clusters. However, both mRNA and protein expression of HDLBP/vigilin is upregulated similarly by intracellular cholesterol loading [27].

The positive correlation between HDL-C and *APOA1* transcript existed among 22 HDL-related genes, while ten gene transcripts (*ABCA1, BMP1, CUBN, HDLBP, LCAT, LDLR, PRKACB, PRKACG, SCARB1 *and *ZDHHC8*) correlated negatively; they included six genes mentioned earlier [14]. Four gene transcripts (CUBN, HDLBP, PRKACB, and PRKACG) negatively correlated with HDL-C in the present study. The significant decrease of *HDLBP* which expression is upregulated by intracellular cholesterol loading [27], thus confirms our earlier suggestion [14] on the decreased level of cholesterol in PBMC at hyperalphalipoproteinemia. Additionally, the decrease of the *CUBN* transcript, which codes the receptor for apoA-I and HDL [25], may result in an increase of HDL level. A decrease in the content of *LCAT* and *SCARB1* transcripts may be suggested to result in the diminished flow of cholesteryl ester to the liver, thus contributing to hyperalphalipoproteinemia [14].

Functional relations between protein products of genes in two clusters include only two crossing genes. The existence of only two nodes may correspond to the limited number of synchronous changes in node activities in both clusters and the possibility of discordant changes of cluster functionality. Functional isolation of two clusters may underlie the possibility of non-coincident changes between the loss of HDL function and the gain of dysfunction [12]. Multiple pleiotropic effects, namely anti-inflammatory activity, vasodilatory function, antiapoptotic activity, antioxidative activity and cholesterol efflux capacity [12], characterize HDL functions. It may be speculated further that therapy targeting these two nodes, for instance by drugs and\or microRNA, may influence both HDL functionality and atherogenesis. Interestingly, one of two nodes, namely *CSF2RB* mRNA, possesses the greatest pleiotropy across six cardiometabolic traits [38].

## Conclusion

Coordinate regulation of cholesterol influx and efflux in PBMC in atherosclerosis-free subjects with widely varied HDL-C level is suggested. The decreased synthesis and transport of cholesteryl ester to the liver may contribute to hyperalphalipoproteinemia. HDL-C increase is associated with the decrease of expression of innate immunity and inflammation genes. Visualization of twenty-two responder genes is suggested to be useful in the validation of HDL functionality and atherogenesis even at the absence of morphologically evident coronary stenosis.

*Acknowledgements*. The reported study was funded by the Russian Foundation for Basic Research according to the research project No. 17-04-00217.

## Conflict of interest statement

The authors declare that they have no conflict of interest.

## List of abbreviations

Chol, cholesterol; FDR, false discovery rate; GWAS, genome wide association study; HDL-C, highdensity lipoprotein cholesterol; LDL-C, low-density lipoprotein cholesterol; PBMC, peripheral blood mononuclear cells; qPCR, quantitative polymerase chain reaction; RCT, reverse cholesterol transport; RT-PCR, real-time polymerase chain reaction; TG, triglyceride.
